# The impact of systematic assessment for adverse events on unscheduled hospital utilization in patients receiving neoadjuvant or adjuvant chemotherapy: A retrospective multicenter study

**DOI:** 10.1002/cam4.4476

**Published:** 2021-12-09

**Authors:** Jwa Hoon Kim, Seyoung Seo, Jee Hyun Kim, Su‐Jin Koh, Yongchel Ahn, Kyung Hae Jung, Jin‐Hee Ahn, Sung‐Bae Kim, Tae Won Kim, Yong Sang Hong, Sun Young Kim, Jeong Eun Kim, Sang‐We Kim, Dae Ho Lee, Jae Cheol Lee, Chang‐Min Choi, Shinkyo Yoon, Jae Ho Jeong, Hwa Jung Kim, Koung Jin Suh, Se Hyun Kim, Yu Jung Kim, Young Joo Min, Jin Ho Baek, Sook Ryun Park

**Affiliations:** ^1^ Department of Oncology Asan Medical Center University of Ulsan College of Medicine Seoul Republic of Korea; ^2^ Division of Oncology Department of Internal Medicine Korea University Anam Hospital Korea University College of Medicine Seoul Republic of Korea; ^3^ Department of Internal Medicine Seoul National University Bundang Hospital Seoul National University College of Medicine Seongnam Republic of Korea; ^4^ Division of Hematology and Oncology Ulsan University Hospital Ulsan Republic of Korea; ^5^ Department of Hematology and Oncology Gangneung Asan Hospital University of Ulsan College of Medicine Gangneung‐si Republic of Korea; ^6^ Department of Clinical Epidemiology and Biostatistics Asan Medical Center University of Ulsan College of Medicine Seoul Republic of Korea; ^7^ Department of Oncology/Hematology Kyungpook National Universtiy Chilgok Hospital School of Medicine Kyungpook National University Daegu Republic of Korea

**Keywords:** adverse event, chemotherapy, emergency room visit, hospitalization

## Abstract

**Background:**

This study was conducted to compare the reported adverse event (AE) profiles and unexpected use of medical services during chemotherapy between before and after the healthcare reimbursement of AE evaluation in patients with cancer.

**Patients and Methods:**

Using the electronic medical record database system, extracted patients with breast, lung, gastric, and colorectal cancers receiving neoadjuvant or adjuvant chemotherapy between September 2013 and December 2016 at four centers in Korea were matched using the 1:1 greedy method: pre‐reimbursement group (*n* = 1084) and post‐reimbursement group (*n* = 1084). Unexpected outpatient department (OPD), emergency room (ER) visit, hospitalization rates, and chemotherapy completion rates were compared between the groups.

**Results:**

The baseline characteristics were well‐balanced between the groups. By chemotherapy cycle, hospitalization (1.8% vs. 2.3%; *p* = 0.039), and ER visit rates (3.3% vs. 3.9%; *p* = 0.064) were lower in the post‐reimbursement group than that in the pre‐reimbursement group. In particular, since cycle 2, ER visit and hospitalization rates were significantly lower in the post‐reimbursement group than those in the pre‐reimbursement group (2.6% vs. 3.3%; *p* = 0.020 and 1.4% vs. 2.0%; *p* = 0.007, respectively), although no significant differences were observed during cycle 1. The OPD visit rates were similar between both groups, regardless of cycles. The post‐reimbursement group had a higher proportion of patients who completed chemotherapy as planned than the pre‐reimbursement group (93.5% vs. 90.1%; *p* = 0.006). Post‐reimbursement group had more AEs reported, including alopecia, fatigue, diarrhea, anorexia, and peripheral neuropathy, during cycle 1 than the pre‐reimbursement group, which significantly decreased after cycle 2.

**Conclusion:**

The introduction of healthcare reimbursement for AE evaluation may help physicians capture and appropriately manage AEs, consequently, decreasing hospital utilization and increasing chemotherapy completion rates.

## Graphical abstract

Physicians frequently underreport or underestimate the incidence and severity of adverse events (AEs) experienced by patients even in clinical trials and real‐world routine clinical practice. AE profiles and unexpected use of medical services during chemotherapy were compared between before and after the approval of the healthcare policy for the reimbursement of AE evaluation in patients with cancer. The introduction of healthcare reimbursement for AE evaluation may help physicians capture and appropriately manage AEs, consequently, decreasing hospital utilization and increasing chemotherapy completion rates.

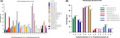

## 1 INTRODUCTION

Monitoring and assessing adverse events (AEs) in patients receiving systemic anticancer treatment are essential in ensuring patient safety and making clinical decisions, such as treatment delay, dose or schedule modification, and treatment discontinuation. Therefore, assessing AEs is a standard procedure in not only clinical trials, but also routine practice and has been performed most commonly using the National Cancer Institute (NCI) common terminology criteria for adverse events (CTCAE), which provides the definition and severity grading of AEs.^1^ The CTCAE includes items of AEs derived from objective data, such as laboratory abnormalities and subjective symptoms experienced by the patients.

Currently, the assessment and reporting of AEs are usually performed by physicians, but it has been reported that physicians frequently underreport or underestimate the incidence and severity of AEs experienced by patients even in clinical trials as well as real‐world routine clinical practice.^2^, ^3^, ^4^ Recognizing this discrepancy in reporting AEs between physicians and patients, the use of patient‐reported outcomes (PROs) is becoming increasingly popular in AE monitoring in clinical trials.^5^ However, the accurate capturing and grading of AEs by physicians are still of paramount importance, and considering its resource‐intensiveness, an effective way in doing that in routine clinical practice needs to be further developed.

In South Korea, as part of the reorganization of patients’ safety‐related medical fees, the assessment of AEs by physicians in patients receiving systemic anticancer agents has become a medical service reimbursed by the National Health Insurance (NHI) since September 2015.

This study was conducted to compare the reported AE profiles and unscheduled hospital visits, including inpatient admissions or visits to the outpatient department (OPD)/emergency room (ER), during chemotherapy between before and after the healthcare reimbursement of AE evaluation in patients with cancer receiving neoadjuvant or adjuvant chemotherapy. To minimize bias from cancer‐associated symptoms, patients with nonmetastatic cancer undergoing standard neoadjuvant or adjuvant chemotherapy after curative‐intent surgery were studied.

## 2 PATIENTS AND METHODS

### 2.1 Study populations

Using the electric medical record (EMR) database system, patients were identified based on the diagnosis of breast, lung, gastric, and colorectal cancers and the administration of neoadjuvant or adjuvant chemotherapy between September 2013 and December 2016 at Asan Medical Center (Seoul, Korea), Seoul National University Bundang Hospital (Seongnam, Korea), Ulsan University Hospital (Ulsan, Korea), and Gangneung Asan Hospital (Gangneung, Korea). Patients who underwent palliative surgery; those who received neoadjuvant or adjuvant concurrent chemoradiation therapy; those who were lost to follow‐up for reasons other than AEs; those who participated in clinical trials, and those whose disease progressed during neoadjuvant or adjuvant chemotherapy were excluded.

In Korea, the healthcare system is implemented under the NHI program, which was began in 1977, and is compulsory by law and is a universal social insurance program that covers the entire population. The Ministry of Health and Welfare oversees the NHI system and its two fundamental institutions: The National Health Insurance Service (NHIS) and the Health Insurance Review & Assessment Service (HIRA). The NHIS serves as the insurer and HIRA conducts claims reviews and quality assessment of healthcare services. Through this system, healthcare providers are required to claim medical services performed by themselves for reimbursement of payments by the NHI, and reimbursement is performed after review by the HIRA. All standard treatments of the neoadjuvant/adjuvant chemotherapy included in this study were covered by NHI during study period.

As the assessment of AEs started to be reimbursed by the NHI since September 2015, patients were classified into two groups: the pre‐reimbursement (September 2013 to August 2015) and post‐reimbursement (January 2016 to December 2016) groups. The exact matching was used along with the 1:1 greedy nearest neighbor algorithm within specified caliper widths based on age (<60 and ≥60 years), sex, cancer type, chemotherapy regimen, and treatment settings (neoadjuvant or adjuvant). The details of chemotherapy regimen according to the cancer type between the pre‐reimbursement and post‐reimbursement groups are shown in the Table S1.

This study was approved by the Institutional Review Board (IRB) of each participating center, and all information was obtained with appropriate IRB waivers.

### 2.2 Clinical data and AEs collection

Clinical data regarding baseline characteristics, treatment, and AEs were retrospectively collected. Past and current medical history included hypertension, diabetes mellitus, tuberculosis, hepatitis, congestive heart failure, coronary artery disease, and chronic obstructive pulmonary disease. AEs were evaluated according to the NCI CTCAE. To assist physicians in capturing and grading AEs and to facilitate claims for reimbursement, most hospitals have introduced systematic toxicity assessment form (STAF) (Figure S1) containing common chemotherapy‐related AE items and severity grading into the EMR system. Besides AEs in STAF, all AEs in the medical records written by physicians were also collected.

### 2.3 Study endpoints and statistical analysis

The primary endpoints were the rate of unexpected utilization of medical services during chemotherapy including unexpected OPD and ER visits, and hospitalization rates. The secondary endpoints included chemotherapy completion rates and dose intensity or dose reduction rates.

Categorical and quantitative data were compared using the chi‐square test or Fisher's exact test and Mann–Whitney *U*‐test, respectively. The unexpected OPD and ER visit and hospitalization rates per patient or chemotherapy cycle were compared between the groups during all chemotherapy periods or according to the treatment period. The treatment periods were divided into the early (“during cycle 1”) and late (“since cycle 2”) periods. The impact of reimbursement for AE evaluation in terms of the unexpected ER visit since cycle 2 was estimated in the subgroup analysis. Two‐sided *p*‐values of less than 0.05 were used to denote statistical significance, and all statistical analyses were performed using Statistical Package for the Social Science (version 23.0; IBM Corp.).

## 3 RESULTS

### 3.1 Patient characteristics

**FIGURE 1:**
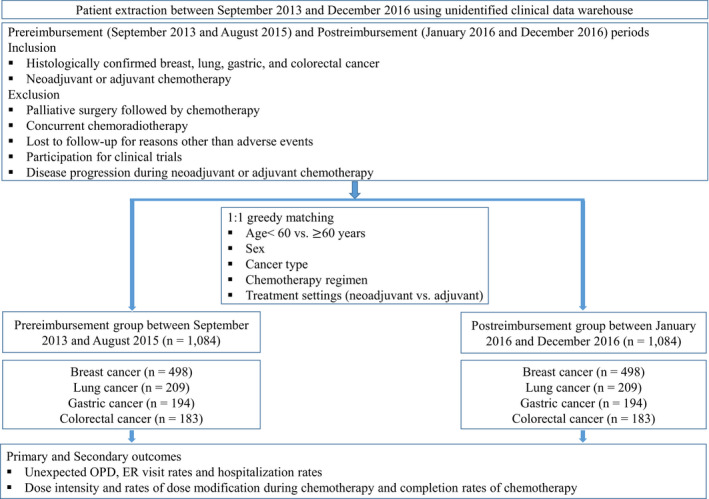
Consort diagram. ER, emergency room; OPD, outpatient department

In this study (Figure 1), 2168 patients with breast, lung, gastric, and colorectal cancers who were treated with neoadjuvant or adjuvant chemotherapy were classified into the pre‐reimbursement (*n* = 1084) and post‐reimbursement (*n* = 1084) groups after exact matching. The median age of the patients was 56 years (range, 17–84 years), and 68.7% of the patients were female. The most common tumor type was breast cancer (*n* = 996, 45.9%), and most patients (*n* = 2153, 99.3%) had Eastern Cooperative Oncology Group Performance Scores (ECOG PS) of 0–1. The STAF was used in 64 patients (5.9%) in the pre‐reimbursement group and 949 patients (87.5%) in the post‐reimbursement group (*p* < 0.001). The baseline characteristics of the patients are presented in Table 1. No significant differences in the baseline characteristics were observed between the two groups, except for a higher proportion of patients with ECOG PS of ≥1 (40.1% vs. 33.3%; *p* < 0.001) in the post‐reimbursement group. Only 15 patients had ECOG PS of 2–3, and no patients had ECOG PS of 4. There were also no significant differences in types of surgery per each cancer type between the two groups (Table S2).

**TABLE 1:** Baseline characteristics of the patients

Characteristics	Pre‐reimbursement group *N* = 1084 (%)	Post‐reimbursement group *N* = 1084 (%)	*p*‐value
Age, years			1.000
<60 years	665 (61.3)	665 (61.3)	
≥60 years	419 (38.7)	419 (38.7)	
Sex			
Male	339 (31.3)	339 (31.3)	1.000
Female	745 (68.7)	745 (68.7)	
Cancer type			1.000
Breast	498 (45.9)	498 (45.9)	
Lung	209 (19.3)	209 (19.3)	
Stomach	194 (17.9)	194 (17.9)	
Colon	183 (16.9)	183 (16.9)	
Pathologic stage			
Breast	*N* = 498 (%)	*N* = 498 (%)	0.821
1	107 (21.5)	97 (19.5)	
2	303 (60.8)	305 (61.2)	
3	85 (17.1)	92 (18.5)	
4	3 (0.6)	4 (0.8)	
Lung	*N* = 209 (%)	*N* = 209 (%)	0.386
1	18 (8.6)	27 (12.9)	
2	93 (44.5)	98 (46.9)	
3	94 (45.0)	81 (38.8)	
4	4 (1.9)	3 (1.4)	
Stomach	*N* = 194 (%)	*N* = 194 (%)	0.702
1	22 (11.3)	18 (9.3)	
2	112 (57.7)	115 (59.3)	
3	53 (27.3)	50 (25.8)	
4	7 (3.6)	11 (5.7)	
Colon	*N* = 183 (%)	*N* = 183 (%)	0.373
1	1 (0.5)	0 (0.0)	
2	66 (36.1)	54 (29.5)	
3	101 (55.2)	113 (62.1)	
4	15 (8.2)	16 (8.8)	
Treatment settings			1.000
Neoadjuvant	491 (45.3)	491 (45.3)	
Adjuvant	593 (54.7)	593 (54.7)	
Postoperative weight change			1.000
Weight loss >10%	112 (10.3)	113 (10.4)	
Past or current medical history^a^			0.688
Present	393 (36.3)	402 (37.1)	
ECOG PS			0.001
0	723 (66.7)	649 (59.9)	
≥1^b^	361 (33.3)	435 (40.1)	
Marriage			0.713
Single	56 (5.2)	71 (6.5)	
Married	904 (83.4)	1002 (92.5)	
Unknown	124 (11.4)	11 (1.0)	

Abbreviations: ECOG PS, Eastern Cooperative Oncology Group Performance Score.

^a^ Medical history included hypertension, diabetes mellitus, tuberculosis, hepatitis, congestive heart failure, coronary artery disease, and chronic obstructive pulmonary disease.

^b^ Only 15 patients had ECOG PS of 2–3, and no patients had ECOG PS of 4—1 patient (0.1%) in the pre‐reimbursement group and 14 patients (1.3%) in the post‐reimbursement group.

### 3.2 Unexpected OPD and ER visit and hospitalization rates

**FIGURE 2:**
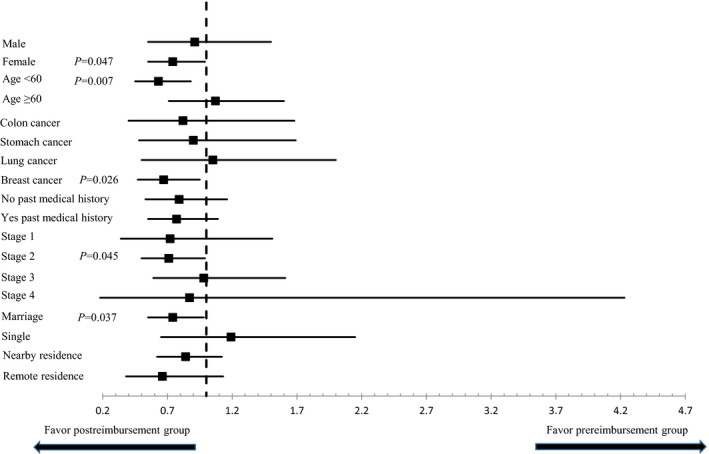
Unexpected emergency room visits since cycle 2 according to the subgroups

Table 2 summarizes the unexpected utilization rates of medical services during chemotherapy per patient. No significant differences in unexpected OPD visit (12.8% vs. 12.5%; *p* = 0.897), ER visit (17.6% vs. 15.2%; *p* = 0.147), and hospitalization (11.2% vs. 10.1%; *p* = 0.443) rates were observed between both groups. Interestingly, when we analyzed by dividing treatment periods into the early (during cycle 1) and late periods (since cycle 2), during cycle 1, no significant differences in the rates of unexpected OPD and ER visits and hospitalization were observed between the groups, but since cycle 2, the post‐reimbursement group was less likely to visit the ER than the pre‐reimbursement group (10.9% vs. 13.6%; *p* = 0.057). In the subgroup analysis, the beneficial effect of reimbursement for AE evaluation on ER visits since cycle 2 was larger in breast cancer patients (odds ratio (OR), 0.67; *p* = 0.026), female patients (OR, 0.74; *p* = 0.047), younger patients less than 60 years (OR, 0.63; *p* = 0.007), patients with earlier stage 1–2 (OR, 0.71; *p* = 0.028), and married patients (OR, 0.74; *p* = 0.037) (Figure 2).

**TABLE 2:** Rates of unexpected utilization of medical services per patient

	Pre‐reimbursement group *N* = 1084 (%)	Post‐reimbursement group *N* = 1084 (%)	*p*‐value
OPD visit	139 (12.8)	136 (12.5)	0.897
ER visit	191 (17.6)	165 (15.2)	0.147
Hospitalization	121 (11.2)	109 (10.1)	0.443
According to the treatment periods			
During cycle 1			
OPD visit	48 (4.4)	47 (4.3)	1.000
ER visit	77 (7.1)	81 (7.5)	0.804
Hospitalization	44 (4.1)	48 (4.4)	0.749
Since cycle 2			
OPD visit	102 (9.4)	96 (8.9)	0.709
ER visit	147 (13.6)	118 (10.9)	0.057
Hospitalization	88 (8.1)	72 (6.6)	0.218

Abbreviations: ER, emergency room; OPD, outpatient department.

Table 3 summarizes the unexpected utilization rates of medical services per chemotherapy cycle. Although no significant difference in OPD visit rates was observed between the two groups (2.7% vs. 2.9%, respectively; *p* = 0.513), the hospitalization rate in the post‐reimbursement group was significantly lower than that in the pre‐reimbursement group (1.8% vs. 2.3%, respectively; *p* = 0.039), and a decreasing trend of ER visit rates was observed in the post‐reimbursement group compared with the pre‐reimbursement group (3.3% vs. 3.9%; *p* = 0.064). Since cycle 2, the ER visit (2.6% vs. 3.3%; *p* = 0.020) and hospitalization (1.4% vs. 2.0%; *p* = 0.007) rates in the post‐reimbursement group were significantly lower than those in the pre‐reimbursement group, although no significant differences in these rates were observed during cycle 1. The OPD visit rates were similar between both groups, regardless of cycles.

**TABLE 3:** Rates of unexpected utilization of medical services per chemotherapy cycle

	Pre‐reimbursement group *N* = 1084 (%)	Post‐reimbursement group *N* = 1084 (%)	*p*‐value
OPD visit	185 (2.7)	207 (2.9)	0.513
ER visit	268 (3.9)	239 (3.3)	0.064
Hospitalization	160 (2.3)	132 (1.8)	0.039
According to the treatment periods			
During cycle 1			
OPD visit	48 (4.4)	47 (4.3)	0.916
ER visit	77 (7.1)	81 (7.5)	0.741
Hospitalization	44 (4.1)	48 (4.4)	0.670
Since cycle 2			
OPD visit	137 (2.4)	160 (2.6)	0.386
ER visit	191 (3.3)	158 (2.6)	0.020
Hospitalization	116 (2.0)	84 (1.4)	0.007

Abbreviations: ER, emergency room; OPD, outpatient department.

### 3.3 Completion rates, dose intensity, and dose modification of chemotherapy

The post‐reimbursement group had a significantly higher proportion of patients who completed chemotherapy as planned than the pre‐reimbursement group (93.5% vs. 90.1%, respectively; *p* = 0.006) (Table 4). No significant differences in dose intensity (*p* = 0.112) and dose modification (*p* = 0.639 for initial dose reduction from cycle 1 and *p* = 0.490 for subsequent dose reduction) were observed between the two groups (Table 4).

**TABLE 4:** Administration of chemotherapy between the groups

	Pre‐reimbursement group *N* = 1084 (%)	Post‐reimbursement group *N* = 1084 (%)	*p*‐value
Total cycles completion as planned	977 (90.1)	1013 (93.5)	0.006
Dose intensity (standard deviation)	0.84 (±0.22)	0.85 (±0.21)	0.112
Dose reduction			
Initially from cycle 1	178 (16.4)	169 (15.6)	0.639
Subsequently	269 (24.8)	284 (26.2)	0.490

### 3.4 AE profiles after cycles 1 and 2 between the two groups

**FIGURE 3:**
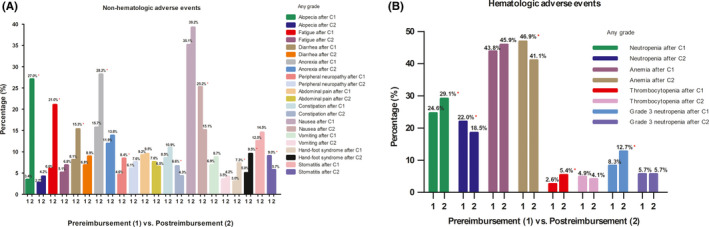
Nonhematologic and hematologic adverse events after cycle 1 (A) and 2 (B) between the pre‐reimbursement and post‐reimbursement groups (≥5% or major). *p < 0.05. ALT, alanine aminotransferase; AST, aspartate aminotransferase

After cycle 1, significantly more nonhematologic AEs were reported in the post‐reimbursement group than in the pre‐reimbursement group—alopecia (27.0% for post‐reimbursement group vs. 3.4% for pre‐reimbursement group; *p* < 0.001), fatigue (21.0% vs. 6.0%; *p* < 0.001), diarrhea (15.3% vs. 8.1%; *p* < 0.001), anorexia (28.2% vs. 15.7%; *p* < 0.001), and peripheral neuropathy (8.4% vs. 4.6%; *p* < 0.001) (Figure 3A). However, these differences decreased after cycle 2: alopecia (4.2% vs. 2.7%; *p* = 0.058), fatigue (6.8% vs. 5.1%; *p* = 0.085), diarrhea (8.9% vs. 6.8%; *p* = 0.067), anorexia (13.8% vs. 11.9%; *p* = 0.178), and peripheral neuropathy (7.6% vs. 6.1%; *p* = 0.173) (Figure 3A). Regarding stomatitis, constipation, and nausea, which occurred numerically more after cycle 1 in the post‐reimbursement group, after cycle 2, their frequency in the post‐reimbursement group decreased more than that in the pre‐reimbursement group: stomatitis (5.7% vs. 9.0%; *p* = 0.003), constipation (4.3% vs. 6.6%; *p* = 0.018), and nausea (15.1% vs. 25.2%; *p* < 0.001) (Figure 3A).

Similar trends of hematologic AEs were observed between both groups; however, numerical differences and changes were modest (Figure 3B). Whereas, after cycle 1, the incidences of all‐grade neutropenia (29.1% vs. 24.6%; *p* = 0.020), thrombocytopenia (5.4% vs. 2.6%; *p* = 0.001), alanine aminotransferase elevation (8.2% vs. 4.9%; *p* = 0.002), and hyperbilirubinemia (2.2% vs. 1.0%; *p* = 0.027) were higher in the post‐reimbursement group than those in the pre‐reimbursement group, their frequency became lower in the post‐reimbursement group (18.5% vs. 22.0% for neutropenia; *p* = 0.037) or similar between both groups after cycle 2. Likewise, the incidence of grade 3 neutropenia was higher in the post‐reimbursement group than that in the pre‐reimbursement group after cycle 1 (12.7% vs. 8.3%; *p* = 0.003) but became similar between the two groups after cycle 2 (Figure 3B).

## 4 DISCUSSIONS

Adverse events during anticancer treatment cover a spectrum of patient symptoms, laboratory values, clinical findings, and radiological examinations. Among them, subjective symptoms are at a higher risk of being underreported by physicians, even when prospectively collected within randomized trials.^2^ In a study evaluating the agreement between 1090 patients receiving cytotoxic chemotherapy for breast or non‐small cell lung cancer and physicians in reporting six chemotherapy‐related AEs (anorexia, nausea, vomiting, constipation, diarrhea, and hair loss) in three randomized trials, for patients who reported toxicity (any severity), underreporting by physicians ranged from 40.7% to 74.4%, and examining only patients who reported “very much” toxicity, underreporting by physicians ranged from 13.0% to 50.0%.^2^ A prospective multicenter study involving 604 patients with breast cancer receiving adjuvant chemotherapy outside a clinical trial has also shown that the frequency and severity of chemotherapy‐related AEs were consistently greater in patient‐reported data than physician‐reported data with a low interrater agreement for most AEs, ranging from 0.10 for anorexia to 0.54 for vomiting (Cohen *κ* statistic).^4^ Interestingly, the discrepancies in AE reporting positively correlated with the number of patients enrolled at each site, suggesting that patient workload affects the discrepancy between physician and patient reporting of AEs.^4^ Considering the clinical practice setting where physicians are challenged by time constraints and high workloads, a better system or tool to facilitate the evaluation of AEs by physicians could decrease these physician–patient discrepancies.^6^

Importantly, AEs during chemotherapy, if not managed appropriately, can often interfere with treatment continuation as planned, reduce patients’ quality of life, and increase healthcare utilization and costs.^7^ Since most chemotherapy‐related AEs are predictable and preventable, if physicians correctly identify and assess AEs, they can be reduced during subsequent cycles through appropriate preemptive management. In this context, we hypothesized that the NHI coverage for AE evaluation improves the capturing of AEs by physicians in routine clinical practice, which has a positive impact on unplanned acute hospital use and proceeding with chemotherapy as planned. Indeed, the results in this study demonstrated that the introduction of healthcare reimbursement for AE evaluation resulted in better capturing of AEs, lower ER visits and unscheduled hospitalization since cycle 2, and a higher chemotherapy completion rate in patients receiving neoadjuvant or adjuvant chemotherapy for breast, lung, colon, and stomach cancers. While physicians reported more AEs after cycle 1 in the post‐reimbursement group than in the pre‐reimbursement group, when the AE profiles between after cycle 1 and cycle 2 were compared in both groups, most AEs reported after cycle 1, including alopecia, fatigue, diarrhea, anorexia, and peripheral neuropathy, were more reduced after cycle 2 in the post‐reimbursement group than in the pre‐reimbursement group. This suggests that physicians might have identified the AEs better after cycle 1 and delivered more proactive management for cycle 2 in the post‐reimbursement group, which can work better, especially in nonhematologic AEs based on patients’ reporting. This favorable impact was also shown in unscheduled utilization of medical services; while unscheduled visits during cycle 1 were not different between both groups, unscheduled ER visits (3.3% vs. 2.6%; *p* = 0.020) and hospitalization rates (2.0% vs. 1.4%; *p* = 0.007) since cycle 2 significantly decreased in the post‐reimbursement group compared with those in the pre‐reimbursement group.

When it comes to the details of protocols of the same chemotherapy regimen, which could be a possible factor contributing to the results, the chemotherapy protocols in terms of doses and schedules were not different between pre‐ and post‐reimbursement groups because they should have been the same as the protocols approved by regulatory authority, South Korea's Ministry of Food and Drug Safety (MFDS), which have been based on the latest version of International and Korean guidelines, including global pivotal trials. The compliance to the approved doses and schedules of chemotherapy is subject to the evaluation by HIRA system in Korea. In addition, since 2011 (before our study period, 2013–2016), the HIRA system has been assessing the quality of cancer care, including adjuvant chemotherapy, in patients who received surgery for major five cancers including gastric, lung, colorectal, breast, and liver cancers to reduce the variability of quality between individual healthcare providers, resulting in more stable and consistent provision of healthcare services nationwide. Based on these, we believe that the protocols of the same chemotherapy regimen were also not different between all four centers.

Studies have reported that proactive approaches to manage chemotherapy toxicity, such as telephone‐based support or electronic symptom monitoring, could improve symptom control and quality of life and decrease ER visits, but these approaches required additional healthcare resources, such as nurses’ telephone calls or counseling outside regular clinic hours, which are significant barriers for widespread implementation in a real‐world setting.^8^, ^9^ However, our results demonstrated that through an appropriate healthcare reimbursement system without resource barriers in hospitals, physicians’ AE evaluation was improved, which led to favorable healthcare service use in the clinical practice. Of note, this better AE reporting system could be, in part, attributed to the use of a systematic assessment tool containing common AE items into the EMR system in each hospital, whereas traditional data collection typically involved unstructured patient interviews. To further improve the quality and comprehensiveness of AE evaluation, not only better physician reports, but also more patient‐involved tools, such as PRO measures, which are increasingly being used in drug development trials, should be widely implemented in routine clinical practice, which could be facilitated through healthcare reimbursement by the NHI. In addition, given the differential beneficial effect of reimbursement on ER visits according to the subgroup (sex, age, cancer type, stage, marriage) in our study (Figure 2), more tailored strategies in AE evaluation and management need to be developed.

This study has the following strengths: it was a large, multicenter study and focused on neoadjuvant or adjuvant chemotherapy for curatively resected nonmetastatic breast, lung, colon, and stomach cancers, minimizing potential confounders related to cancer‐related symptoms. In addition, since the patients received chemotherapy in routine clinical practice and not in a prospective clinical trial mandating standardized AE reporting, the study results reflect real‐world practice. However, this study has some limitations. First, although we suggested intensified symptom management by physicians as a result of improved AE assessment as a mechanism for clinical benefits based on the results of other studies,^10^, ^11^ this study did not evaluate medications or treatments related to supportive care. Secondly, we did not perform cost‐utility analysis, which could be a relevant topic for future research to justify further investment from healthcare services. Third, in general, supportive care might have improved over time, which might have affected the incidence and severity of AE or unscheduled use of medical services in a comparison between two different periods (September 2013 to August 2015 in the pre‐imbursement group vs. January 2016 to December 2016 in the post‐reimbursement group) in our study. Specifically, during the study periods (2013–2016), the prophylactic use of long‐acting G‐CSF in patients with breast cancer receiving neoadjuvant/adjuvant AC (anthracycline plus cyclophosphamide)‐containing chemotherapy became eligible for reimbursement by the NHI from September 2016 in Korea. However, the long‐acting G‐CSF could be already used in these patients if they agreed with medical expenses uncovered by the NHI since July 2014. Although data regarding the frequency of the prophylactic use of long‐acting G‐CSF between the two periods was not available in our study, there were no significant differences in the use of conventional G‐CSF (33.5% vs. 33.3%), any grades of neutropenia (40.5% vs. 34.9%; *p* = 0.101), and febrile neutropenia (14.1% vs. 13.7%; *p* = 0.854) between the pre‐reimbursement and post‐reimbursement group in breast cancer patients. Otherwise, there were no significant changes of health insurance systems between the two periods in our study.

In conclusion, our analysis showed that the introduction of healthcare reimbursement by the NHI for AE evaluation may have a positive impact on physicians’ AEs capturing, and acute hospital utilization and chemotherapy completion in patients receiving adjuvant chemotherapy. Our findings highlight the importance of AE evaluation and the effect of healthcare reimbursement policy on the quality of oncology clinical practice.

## CONFLICT OF INTEREST

All authors have no conflict of interest to declare.

## AUTHOR CONTRIBUTIONS

study concepts: Sook Ryun Park; patient management: Seyoung Seo, Jee Hyun Kim, Su‐Jin Koh, Yongchel Ahn, Kyung Hae Jung, Jin‐Hee Ahn, Sung‐Bae Kim, Tae Won Kim, Yong Sang Hong, Sun Young Kim, Jeong Eun Kim, Sang‐We Kim, Dae Ho Lee, Jae Cheol Lee, Chang‐Min Choi, Shinkyo Yoon, Jae Ho Jeong, Hwa Jung Kim, Koung Jin Suh, Se Hyun Kim, Yu Jung Kim, Young Joo Min, Jin Ho Baek, and Sook Ryun Park; Data analysis and interpretation of results: Jwa Hoon Kim, Seyoung Seo, and Sook Ryun Park; statistical analysis: Jwa Hoon Kim, Seyoung Seo, and Sook Ryun Park; manuscript writing: Jwa Hoon Kim, Seyoung Seo, and Sook Ryun Park; manuscript reviewing: Jwa Hoon Kim, Seyoung Seo, Jee Hyun Kim, Su‐Jin Koh, Yongchel Ahn, Kyung Hae Jung, Jin‐Hee Ahn, Sung‐Bae Kim, Tae Won Kim, Yong Sang Hong, Sun Young Kim, Jeong Eun Kim, Sang‐We Kim, Dae Ho Lee, Jae Cheol Lee, Chang‐Min Choi, Shinkyo Yoon, Jae Ho Jeong, Hwa Jung Kim, Koung Jin Suh, Se Hyun Kim, Yu Jung Kim, Young Joo Min, Jin Ho Baek, and Sook Ryun Park.

## ETHICAL APPROVAL

This study was approved by the Institutional Review Board of each participating center, and the requirement of informed consent has been waived because of the retrospective nature of this study. This study was conducted in accordance with the Declaration of Helsinki and Good Clinical Practice.

## Supporting information

Fig S1

Table S1‐S2

## Data Availability

The datasets generated during and/or analyzed during this study are available from the corresponding author (SRP) upon reasonable request. The data are not publicly available due to restriction 'them containing information that could compromise research participant privacy/consent.
