# Supplementary figures and images for: The impact of systematic assessment for adverse events on unscheduled hospital utilization in patients receiving neoadjuvant or adjuvant chemotherapy: A retrospective multicenter study
Source: Cancer Med. 2021 Dec 9;11(3):705–14. doi: 10.1002/cam4.4476 (PMC8817089; doi:10.1002/cam4.4476)

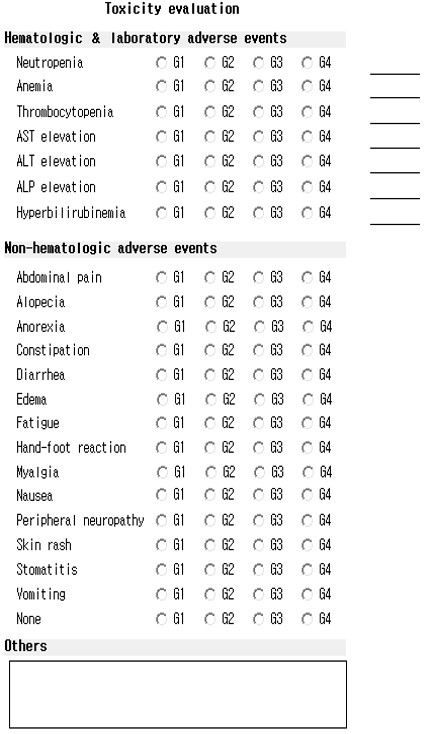

Supplement: Supplementary file 1 — Fig S1 [file CAM4-11-705-s002.jpg]
