# Supplementary material for: The impact of systematic assessment for adverse events on unscheduled hospital utilization in patients receiving neoadjuvant or adjuvant chemotherapy: A retrospective multicenter study
Source: Cancer Med. 2021 Dec 9;11(3):705–14. doi: 10.1002/cam4.4476 (PMC8817089; doi:10.1002/cam4.4476)
Supplement: Supplementary file 2 — Table S1‐S2 [file CAM4-11-705-s001.docx]

**Supplementary Table 1. Chemotherapy regimens according to the cancer type between the pre-reimbursement and post-reimbursement groups**

|  | **Pre-reimbursement group**  **N = 1,084 (%)** | **Post-reimbursement group**  **N = 1,084 (%)** | ***p*-value** |
| --- | --- | --- | --- |
| **Breast cancer** | N = 498, (%) | N = 498, (%) | 1.000 |
| Anthracycline plus cyclophosphamide containing chemotherapy | 435 (87.3) | 435 (87.3) |  |
| Taxane plus cyclophosphamide | 63 (12.7) | 63 (12.7) |  |
| **Lung cancer** | N = 209, (%) | N = 209, (%) | 0.994 |
| Vinorelbine plus cisplatin | 118 (56.5) | 117 (56.0) |  |
| Paclitaxel plus carboplatin/cisplatin | 91 (43.5) | 92 (44.0) |  |
| **Stomach cancer** | N = 194, (%) | N = 194, (%) | 0.815 |
| Capecitabine plus oxaliplatin | 48 (24.7) | 50 (25.8) |  |
| S-1 | 146 (75.3) | 144 (74.2) |  |
| **Colon cancer** | N = 183, (%) | N = 183, (%) | 1.000 |
| 5-FU/capecitabine plus oxaliplatin | 152 (83.1) | 152 (83.1) |  |
| 5-FU or capecitabine | 31 (16.9) | 31 (16.9) |  |

**Supplementary Table 2. Types of surgery between pre-reimbursement and post-reimbursement groups**

|  | **Pre-reimbursement group**  **N = 1,084 (%)** | **Post-reimbursement group**  **N = 1,084 (%)** | ***p*-value** |
| --- | --- | --- | --- |
| **Breast cancer** | N = 498, (%) | N = 498, (%) | 0.149 |
| Breast conserving surgery | 301 (60.4) | 274 (55.2) |  |
| Modified radical mastectomy | 197 (39.6) | 222 (44.8) |  |
| Unknown | 0 (0.0) | 2 (0.4) |  |
| **Lung cancer** | N = 209, (%) | N = 209, (%) | 0.567 |
| Wedge resection or segmentectomy | 10 (4.8) | 6 (2.9) |  |
| Lobectomy | 195 (93.3) | 198 (94.7) |  |
| Pneumonectomy | 4 (1.9) | 5 (2.4) |  |
| **Stomach cancer** | N = 194, (%) | N = 194, (%) | 0.318 |
| Subtotal gastrectomy | 183 (94.3) | 178 (91.8) |  |
| Total gastrectomy | 11 (5.7) | 16 (8.2) |  |
| **Colon cancer** | N = 183, (%) | N = 183, (%) | 0.136 |
| Total colectomy or hemicolectomy | 105 (57.4) | 87 (47.5) |  |
| Anterior resection | 74 (40.4) | 93 (50.8) |  |
| Unknown | 4 (2.2) | 3 (1.6) |  |
